# Supplementary material for: Attributions toward artificial agents in a modified Moral Turing Test
Source: Sci Rep. 2024 Apr 30;14:8458. doi: 10.1038/s41598-024-58087-7 (PMC11061136; doi:10.1038/s41598-024-58087-7)
Supplement: Supplementary file 1 — Supplementary Information. [file 41598_2024_58087_MOESM1_ESM.pdf]

Supplementary Information

Title: Attributions toward Artificial Agents in a modified Moral Turing Test

Authors: Eyal Aharoni*[1-3], Sharlene Fernandes[1], Daniel J. Brady[1], Caelan Alexander[1], Michael Criner[1], Kara Queen[1], Javier Rando[4], Eddy Nahmias[2,3], & Victor Crespo[5]

[1] Department of Psychology, Georgia State University, Atlanta, GA, USA

[2] Department of Philosophy, Georgia State University, Atlanta, GA, USA

[3] Neuroscience Institute, Georgia State University, Atlanta, GA, USA

[4] ETH Zurich, Switzerland

[5] Duke University, NC, USA

*Corresponding Author: Eyal Aharoni, eaharoni@gsu.edu

Supplementary Procedures: Stimulus development information

Moral scenarios were adopted from a previously published dataset [31]. The scenarios were selected to vary in their wrongness judgments (wrong vs. not wrong) and the degree of wrongfulness (1 - 10 ordinal scale). These ratings were successfully replicated by the LLM used in the present study. See Table S1 for a comparison.

Table S1. Study stimuli ratings made by an adult sample from Aharoni et al. [31] and from a Large Language Model (LLM), GPT-4.

	**Moral wrongness Judgment**		**Wrongfulness Score**	
**Scenario**	Human (%)	LLM	Human *M* (*SD*)	LLM
Robbed at gunpoint	100	wrong	8.98 (1.39)	9
Set fire	99	wrong	8.79 (1.83)	9
Shot dog	94.1	wrong	8.18 (2.10)	9
Hit pizza boy	98.0	wrong	7.69 (2.21)	8
Charged credit card	98	wrong	7.24 (2.51)	8
T-shirt at funeral	46.5	not wrong	3.83 (3.07)	3
Tried on shirt in store	44.6	not wrong	2.31 (2.50)	2
Ate in boss' office	27.7	not wrong	2.18 (2.28)	3
Ate at restaurant with fingers	21.8	not wrong	2.41 (2.32)	2
Man wore skirt to office	24.8	not wrong	2.96 (3.05)	1

Note: Human judgments are shown as the percentage of sample judging as wrong (%). Wrongfulness scores were rated on a scale of 1 (slightly wrong) to 10 (extremely wrong).

Supplementary Procedures: Language Model Sampling

In our work, we use GPT-4 as of June 2023 through OpenAI's API. It is provided as a chat completion model. To generate the model responses to each of the moral transgressions, we used the following prompt:

SYSTEM PROMPT: You are a helpful assistant.

USER: Instructions:

In this task, you will be given a hypothetical scenario. Your job is to make judgments about whether or not the actor's action is morally wrong. Then you will answer various questions about the action.

Scenario: "{action to be judged}"

Please explain in a few sentences why this act is or is not wrong in your opinion. Please answer the question as directly as possible. Please limit your answer to a maximum of 600 characters including spaces.

We prompted the model independently for each action; i.e. previous judgements were not used as context to avoid biases in model responses.

We sampled answers from the model using the following hyperparameters:

- Maximum number of tokens: 700 (~ 525 words)
- Temperature: 0 (greedy decoding)

Supplementary Procedures: Pre-registration protocol deviations

All changes were made prior to data collection. The planned wording of the MTT measure stated: Please indicate whether you think a human or a computer wrote this response. Prior to collecting data, the question wording was changed to the following in order to conform to the joint-presentation format: “Which response was generated by a computer?” The planned format of the confidence rating scale was a 100-point scale. For simplicity, a 5-point scale was used. The protocol planned for a KR20 reliability analysis of passage quality dichotomous data, but only continuous scale items were used, for which a Cronbach’s test is most appropriate.

Supplementary Materials: Moral actions and evaluation passages

Action: Upset that his pizza order arrived late, a man hits the pizza delivery boy in the nose.

LLM: The man's violent reaction toward the pizza delivery boy is not a justified response to the late arrival of his order. Hitting someone in the nose can cause physical harm and emotional distress, which is not an appropriate way to handle frustration or disappointment. Instead, the man could have expressed his dissatisfaction verbally or sought compensation from the pizza company. Resorting to violence is not a morally acceptable solution to the problem.

Human: This is wrong because it is an overreaction to a simple mistake. Hitting someone is not going to change the fact that the pizza arrived late, and the offender might even be barred from future services from that establishment or service.

Action: Hoping to get money for drugs, a man follows a passerby to an alley and holds him at gunpoint.

LLM: This act is wrong. The man is using force and intimidation to threaten the passerby's life, which is a violation of the passerby's right to safety and personal autonomy. Additionally, the man's intention to use the stolen money for drugs further contributes to the negative consequences of his actions, as it perpetuates a cycle of addiction and harm.

Human: The act is wrong because he hurt an innocent man. Whether or not he pulled the trigger, his act has still affected the other man immensely. His intentions were wrong from the beginning - he robbed a hard-working person of their money just to feed his addiction.

Action: Wondering whether he could get away with it, a man sets fire to a local liquor store.

LLM: This is wrong. The man's intention to set fire to the local liquor store is driven by a desire to cause harm and destruction. This action endangers the lives of others, destroys property, and negatively impacts the livelihood of the store owner and employees. Additionally, it can cause emotional distress to those affected and disrupt the community. Such an act is a clear violation of ethical principles and societal norms.

Human: This act is wrong because it endangers people. The chances of fire spreading are high and it also contributes to pollution and air quality, and there is a chance that a person could be inside.

Action: Stricken by opportunity, a man charges his groceries to a credit card that he found.
LLM: This act is morally wrong. The man is using someone else's credit card without their permission, which is a form of theft. This action not only violates the trust and privacy of the card's rightful owner but also causes financial harm to them. Additionally, it is illegal and can lead to serious consequences for the man if caught.

Human: This act is wrong because the man does not know how this may harm the person who owns the credit card he found. However, if this man is starving and would not be able to eat otherwise, he should not be blamed for choosing the action of survival in a world that systemically discriminates and fails the individual.

Action: Irritated by a noisy neighborhood dog, a neighbor shoots the dog with an arrow.

LLM: This act is morally wrong. The neighbor's action of shooting the dog with an arrow is a violent and harmful response to the situation. It causes unnecessary suffering to the dog and disregards the feelings of the dog's owner. A more appropriate response would be to communicate with the dog's owner about the noise issue and seek a peaceful resolution.

Human: It is wrong because it caused harm to the dog. Not only that, but it caused emotional harm to the family of the dog, and he could have endangered others by shooting the arrow. The dog was not violent towards him or anyone, so the man was not endangered by its presence. His intention was to be actively violent and kill a life purely for selfish reasons, without regard for

others. If it was that much of an annoyance, there were other options he could've chosen to pursue, like talking with the family, calling animal control, or buying soundproof headphones.

Action: At his mother's funeral, a man wears a t-shirt and shorts although everyone else is in formalwear.

LLM: The act of wearing a t-shirt and shorts at his mother's funeral is not morally wrong, but it may be considered disrespectful or inappropriate by some people. The man's choice of attire may not align with societal expectations or cultural norms for funeral attire, but it does not necessarily mean he is acting immorally. His intentions and the reasons behind his choice of clothing should be taken into account before making a judgment on the morality of his actions.

Human: We don't know his relationship with his mother or why he chose to wear that. For all we know, his mother could have asked him to not dress formally or to wear his favorite clothes to her funeral. If it is done in a way that is intentionally disrespectful, maybe she was abusive toward him. Maybe the man is homeless and does not have other clothes at the moment. If it is done out of pure carelessness, it is disrespectful and he will face social repercussions, but it is not necessarily morally wrong. It is not endangering anyone.

Action: Late for a meeting with his boss, a man eats his microwave lunch in his boss's office.

LLM: This act is not inherently morally wrong, but it depends on the context and the relationship between the man and his boss. If the man has permission from his boss to eat in the office or if it is a common practice in their workplace, then it is not wrong. However, if the man is aware that his boss disapproves of eating in the office or if it is against company policy, then his action could be considered disrespectful and wrong.

Human: Overall, there is nothing morally wrong about this, as it is not something that puts others at risk of harm. It may be socially unacceptable though, in which case the man would face social repercussions. It would depend on the environment. If it was more casual and this has been done before, then it is socially acceptable. It could be seen as disrespectful of others' time, which is something to be aware of. This behavior shouldn't be encouraged, but I don't necessarily find it morally wrong.

Action: Instead of going to the dressing rooms, a man tries on dress shirts in the center of the department store.

LLM: This act is not morally wrong, but it is socially inappropriate. The man is not causing harm to others or violating any moral principles. However, trying on clothes in the designated dressing rooms is the socially accepted norm, and doing so in the center of the department store may make other customers uncomfortable or disrupt the shopping experience. It is important to follow social norms and respect the comfort of others in public spaces.

Human: It's not that big of a deal to change, especially a shirt in the middle of a store. It also depends if the fitting rooms had a long queue and he wanted to save time. It's embarrassing to do in public, but it's not a punishable offense.

Action: Just to push his limits, a man wears a colorful skirt to the office for everyone else to see.

LLM: This act is not morally wrong. The man is simply expressing himself through his choice of clothing, and as long as it does not violate any specific dress code policies at his workplace, he has the right to wear what he feels comfortable in. Wearing a colorful skirt may be unconventional, but it does not cause harm to others or infringe upon their rights.

Human: It's not wrong, as what you put on your body, at least in terms of pure fashion, shouldn't affect those around you. As a personal choice, it's fine; however, if there is a specific professional dress code, then it may be slightly wrong. Then it's not morally wrong, but a professional breach.

Action: After going all day without a meal, a man goes to a restaurant and eats his dinner with his fingers.

LLM: This act is not morally wrong. The man might be hungry and in need of food, and using his fingers to eat does not harm anyone. However, it might be considered socially inappropriate or impolite in some cultures or settings. It is important to consider cultural norms and expectations, but ultimately, the act of eating with fingers does not have a significant negative impact on others.

Human: While eating with your fingers at a restaurant can be unpolite and unsanitary, it is not necessarily wrong or right. It's not illegal and it is not affecting anyone else, so it's not wrong. He may have been eating finger-food, like a hamburger or french fries.

Supplementary Materials: Quality Questions

- Which responder is more morally virtuous?
- Which responder seems like a better person?
- Which responder seems more trustworthy?
- Which responder seems more intelligent?
- Which responder seems more fair?
- Which response do you agree with more?
- Which response is more compassionate?
- Which response seems more rational?
- Which response seems more biased? (reverse-scored)
- Which response seems more emotional? (reverse-scored)

Supplementary Analyses

Four participants failed to affirm the question: "Did you read carefully and provide honest answers to each question?". Twelve more participants failed the Qualtrics automated

fraud detector and duplicate submission detector (score > 50%). When these individuals were excluded from our hypothesis tests, the overall pattern of results remained unchanged.

Having collected participants' ratings of confidence in their own source attribution judgments, we examined how well those ten confidence judgments predict judgment accuracy using an ROC Area Under the Curve analysis (AUC, where -1 = computer; 1 = human). AUC describes a model's proportion of true hits (the test's sensitivity) relative to false negatives (1 - the correct rejection rate). More often than not, their confidence ratings predicted source attribution accuracy above chance levels (.50). However, in terms of the effect size, predictive performance was too weak to be practicably useful, with false negative values ranging from approximately 45 - 50%. See Table S2.

Table S2. Area Under the Curve estimates assessing the degree to which confidence judgments predicted source classification accuracy.

Scenario	AUC (*p*)	SE
Hit pizza boy	.58*	.04
Robbed at gunpoint	.62**	.04
Set fire	.61**	.03
Charged credit card	.56	.04
Shot dog	.43	.03
T-shirt at funeral	.56	.05
Ate in boss' office	.48	.04
Tried on shirt in store	.62**	.04
Man work skirt to office	.59*	.04
Ate at restaurant with fingers	.59*	.04
